# Patient self-inflicted lung injury associated pneumothorax/pneumomediastinum is a risk factor for worse outcomes of severe COVID-19: a case-control study

**DOI:** 10.1038/s41598-024-66229-0

**Published:** 2024-07-04

**Authors:** Zhigui Cai, Xingxing Guo, Xing Lv, Yunfu Wu, Xiaona Niu, Liqiang Song

**Affiliations:** 1 Department of Pulmonary and Critical Care Medicine, Xijing Hospital, Air Force Medical University, No. 169, Changle West Road, Xi’an, 710032 Shaanxi China; 2 Department of Cardiology, Tangdu Hospital, Air Force Medical University, Xi’an, China

**Keywords:** COVID-19, Patient self-inflicted lung injury, Pneumothorax, Pneumomediastinum, Risk factors, Respiratory tract diseases

## Abstract

We aimed to determine the clinical characteristics of patient self-inflicted lung injury (P-SILI)-associated pneumothorax/pneumomediastinum, to reveal its risk factors, and to assess its impact on severe COVID-19 cases. In total, 229 patients were included in this case-control study. They were randomly divided into either the case group or the control group as per the inclusion and exclusion criteria. The two groups were further analyzed to reveal the risk factors of spontaneous pneumothorax/pneumomediastinum (SP/P). Finally, risk factors for death were analyzed in the case group and the relationship between death and SP/P was also analyzed among all patients. The mean age of patients was 59.69 ± 17.01 years, most of them were male (74.2%), and 62.0% of them had comorbidities upon admission. A respiratory rate higher than 30 BPM was a risk factor for SP/P (OR 7.186, 95% CI 2.414–21.391, *P* < 0.001). Patients with delayed intubation due to early application of HFNC or NIV had a higher mortality rate when they developed SP/P (*P* < 0.05). Additionally, advanced age increased the risk of death (*P* < 0.05). Finally, SP/P may be a risk factor for death among patients with severe COVID-19 (OR 2.047). P-SILI occurs in severe COVID-19 with acute respiratory failure. It is necessary to identify the risk factors of P-SILI, the indicators of severe P-SILI, and the preventive measures.

## Introduction

Since the outbreak of coronavirus disease 2019 (COVID-19), the treatment of severe and critical patients has remained a clinical challenge^1^. Despite of the admisistartion of respiratory support, antiviral drugs, and immunomodulators, it is still highly likely for the patients with COVID-19 to develop acute respiratory distress syndrome (ARDS), and eventually, invasive ventilation and ECMO are needed^1^.

Amounting evidence has shown that patient self-inflicted lung injury (P-SILI), which was firstly proposed by Laurent Brochard and colleagues in 2017^2^, may aggravate severe COVID-19. P-SILI refers to lung injury in non-intubated patients with acute respiratory failure and high respiratory drive. This type of lung injury is similar to ventilator-induced lung injury (VILI) in mechanically ventilated patients. Accordingly, it is believed that early intubation should be considered to avoid P-SILI^3,4^. However, some experts argue the existance of P-SILI in clinical practice and claim that early intubation may lead to unnecessary complications and waste of medical resources^5–7^.

Many studies have shown that patients with severe ARDS have augmented respiratory drive, as shown by increased tidal volume and respiratory rate^8,9^. Furthermore, late intubation was associated with increased mortality in patients with ARDS^10,11^. These findings indirectly suggest that P-SILI may actually exist and can deteriorate ARDS.

During the treatment of COVID-19 patients, we have ever identified two cases with spontaneous pneumothorax but without chronic lung disease. Our findings were further supported by several case reports and case series about spontaneous pneumothorax/pneumomediastinum (SP/P) in non-intubated COVID-19 patients. However, the predominant pathology in deceased patients with COVID-19 was diffuse alveolar damage (DAD)^12^. Microscopically, DAD is divided into two main phases.The acute phase is featured with vascular congestion with alveolar septal edema, fibrinoid exudates within the alveolar space, and hyaline membranes. In the organizing phase of DAD, the alveolar interstitium is replaced by fibroblastic tissue, and the alveolar septae are covered by reactive/reparative type II pneumocyte hyperplasia. In general, COVID-19 is mainly characterized by exudation, consolidation, and proliferation. Presumably, P-SILI causes the alveolar rupture and leads to SP/P, one of the most severe form of lung injury associated with P-SILI.

We aimed to conduct a case-control study to determine the clinical characteristics of P-SILI-associated pneumothorax/pneumomediastinum, to identify its risk factors, and to measure its impact on severe COVID-19 cases. Our findings can improve the prevention and treatment of severe COVID-19.

## Materials and methods

### Search strategy

A systematic search was performed in “PubMed”, “Web of Science”, “Embase”, “Scopus”, and “Google Scholar” from December 1, 2019 to May 31, 2022. “COVID-19”, “severe”, “SARS-COV-2”, “Pneumothorax”, “Pneumomediastinum”, and “mediastinal emphysema” were used as the keywords. Case reports and case series about pneumothorax/pneumomediastinum in non-intubated COVID-19 patients were included. We downloaded the full-text version and screened the subjects as per the exclusion criteria and deleted the duplicated cases.

Inclusion criteria were as follows: 1-patients were classified as severe COVID-19 cases according to the guidelines of World Health Organization, and present with SP/P episodes after SARS-CoV-2 infection; 2-at least one case in the series reported a severe COVID-19 patient with concurrent SP/P. Exclusion criteria were as follows: patients underwent intubation before SP/P or the article did not report any clinical data about the patients.

Finally, 73 articles with 119 patients were included in our case-control study. The basic information of included studies is presented in Supplementary Table 1. In addition, 2 patients from Wuhan Huoshenshan COVID-19 hospital and Xi’an People’s COVID-19 hospital who met the inclusion criteria were also included.

### Study design

**Figure 1:**
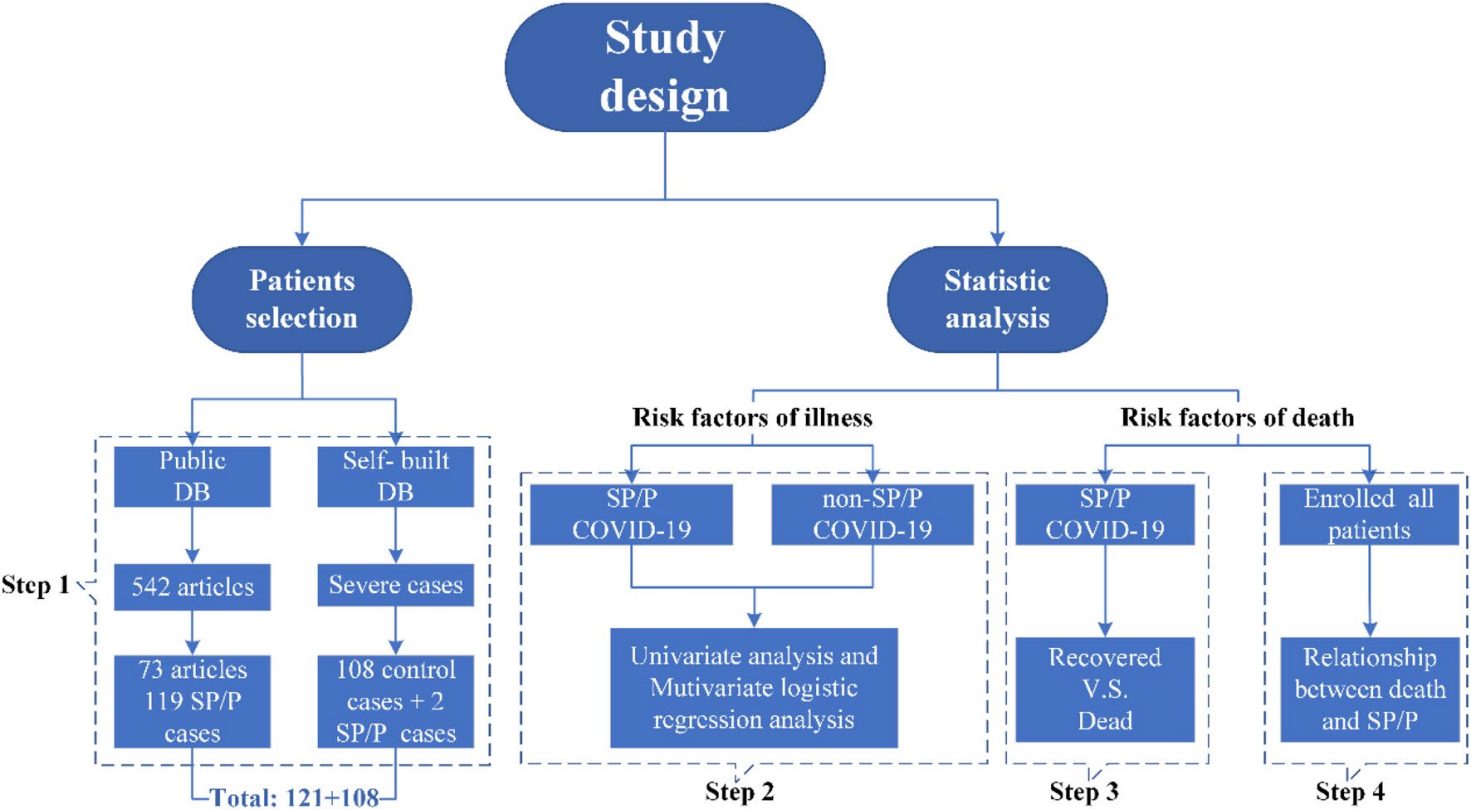
Flowchart. A total of 229 non-intubated COVID-19 patients with spontaneous pneumothorax/pneumomediastinum were identified and included in our case-control study, which was in four steps.

This case-control study was conducted in four steps (Fig. 1). First, 121 patients with pneumothorax/pneumomediastinum were defined as the case group. In addition, patients who were classified as having severe cases of COVID-19 according to the guidelines of the World Health Organization, but did not undergo NIV/MV or experience of SP/P were also searched in a self-built database. Finally, 108 patients were included in the control group.

Then, the two groups were compared to explore the risk factors of SP/P. Next, risk factors for death were analyzed in the case group. There were a total of 121 cases, of which 64 were reported as recovery and defined as group 1, 38 were reported as death and defined as group 2, and 19 were excluded due to the lack of clear outcome reports. Last, the relationship between death and SP/P was also analyzed among all patients.

### Data collection

Three researchers (ZG.C., YF.W., and X.L.) independently reviewed the full-text version of articles to identify studies that evaluated the risk factors, onset, and outcomes of SP/P in patients with COVID-19. Any disagreements were resolved by discussion or by another senior consultant (LQ.S.). The extracted data were pooled in a standardized Excel (Microsoft Corporation) file.

The data containing age, sex, comorbidities, smoking status, oxygen saturation, respiratory rate, respiratory support strategy, outcome, time from symptom onset to severe COVID-19 (days), the interval from symptom onset to SP/P (days), indicators of physical examination, hematologic indices, and C-reactive protein were collected.

### Statistical analysis

Analyses were conducted using SPSS 23 for Windows (SPSS Inc, IBM). Categorical variables were presented as percentages and continuous variables were presented as mean ± standard deviation. Missing values were supplemented through missing value analysis. The difference in qualitative variables was assessed using a Chi-squared test or Fisher test. Factors that were significant in univariate analysis were included in the multivariate logistic regression analysis. Odds ratios (ORs) and 95% confidence intervals (95% CIs) were calculated for factors associated with SP/P. Restricted cubic splines (RCS) were used to describe the nonlinear relationship between variables and outcomes. A *P*-value less than 0.05 was considered significant, but variables with a *P*-value less than 0.10 were included in univariate logistic regression analysis.

### Ethics approval and consent to participate

All methods were performed in accordance with the guidelines and regulations contained in the Declaration of Helsinki. This study was approved by the Medical Ethics Committee of the First Affiliated Hospital of the Air Force Medical University (KY20222118-F-1). Since no intervention was made for enrolled patients and only real-world data were collected, the informed consent was not essential.

## Results

### Demographics and clinical characteristics

The characteristics of all 229 patients were presented in Table 1 and Supplementary Table 2. The mean age of patients was 59.69 ± 17.014 years, most of them were male (74.2%), and 62.0% of the patients had comorbidities upon admission. The time from symptom onset to severe disease in the SP/P COVID-19 group was shorter than that in the non-SP/P COVID-19 group (*P* < 0.001). There was no significant difference between the SP/P COVID-19 group and the non-SP/P COVID-19 group in smoking status, oxygen saturation, blood pressure, routine hematologic indices, C-reactive protein, and outcomes (*P* > 0.05).

**Table 1:** Demographics and clinical characteristics of patients.

Clinical variable	Total cases (N = 229)	SP/P COVID (N = 121)	Non-SP/P COVID (N = 108)	*P* value
Age (years) mean ± SD△	59.69 ± 17.014	52.02 ± 16.551	68.28 ± 12.983	***P* < 0.001**
Sex△				***P* < 0.001**
Male (%)	74.2%	86.0%	61.1%	
Female (%)	25.8%	14.0%	38.9%	
Comorbidities△				***P* < 0.001**
No n (%)	87 (38.0%)	64 (52.9%)	23 (21.3%)	
Yes n (%)	142 (62.0%)	57 (47.1%)	85 (78.7%)	
Asthma n (%)		6 (10.5%)	1 (1.2%)	
Chronic bronchitis n (%)		1 (1.8%)	5 (5.9%)	
COPD n (%)		3 (5.3%)	8 (9.4%)	
Hypertension n (%)		34 (59.6%)	57 (67.1%)	
Coronary heart disease n (%)		4 (7.0%)	11 (12.9%)	
Diabetes n (%)		14 (24.6%)	21 (24.7%)	
Nephropathy n (%)		6 (10.5%)	7 (8.2%)	
Others n (%)		31 (54.4%)	30 (35.3%)	
Smoking status△				*P* = 0.929
No (%)	90.7%	90.9%	90.6%	
Yes (%)	9.3%	9.1%	9.4%	
Oxygen saturation				*P* = 0.078
≥ 93% (%)	25.8%	20.7%	30.8%	
< 93% (%)	74.2%	79.3%	69.2%	
Systolic pressure (mmHg) mean ± SD	132.21 ± 22.257	131.61 ± 21.300	132.89 ± 23.364	*P* = 0.666
Diastolic pressure (mmHg) mean ± SD	78.51 ± 14.597	77.13 ± 13.919	80.05 ± 15.239	*P* = 0.132
Pulse (BPM) mean ± SD	94.03 ± 18.109	96.85 ± 19.615	90.86 ± 15.752	***P* < 0.050**
Respiratory rate (BPM) mean ± SD	25.32 ± 7.212	27.58 ± 7.584	22.79 ± 5.837	***P* < 0.001**
Temperature (℃) mean ± SD	37.21 ± 0.891	37.54 ± 0.929	36.84 ± 0.679	***P* < 0.001**
Respiratory support△				***P* < 0.001**
Conventional oxygen therapy (%)	47.3%	67.8%	23.8%	
Noninvasive respiratory support (%)	52.7%	32.2%	76.2%	
WBC (× 10^9^/L) mean ± SD	10.94 ± 7.762	10.56 ± 8.236	11.35 ± 7.208	*P* = 0.442
Lymphocyte (× 10^9^/L) mean ± SD	0.82 ± 0.547	0.85 ± 0.560	0.79 ± 0.531	*P* = 0.413
C-reactive protein (mg/L) mean ± SD				*P* = 0.633
< 20 (%)	23.6%	22.3%	25.0%	
≥ 20 (%)	76.4%	77.7%	75.0%	
Time from symptom onset to severe status (days) mean ± SD	13.49 ± 11.566	10.48 ± 9.513	16.86 ± 12.722	***P* < 0.001**
Outcome△				*P* = 0.088
Recovered (%)	56.8%	62.7%	51.0%	
Dead (%)	43.2%	37.3%	49.0%	

Except for “△”, other indicators are subject to missing value analysis.

Bold is that the *P*-value represents significant difference, i.e., *P<0.05*.

### Univariate analysis

Univariate analyses were performed (Table 2 and Supplementary Table 3) to identify the risk factors of illness and death. Respiratory rate (OR 8.085, 95% CI 3.263–20.033) and oxygen saturation (OR 1.764, 95% CI 0.969–3.211) were associated with an increased risk of SP/P. Advanced age, female gender, comorbidities, non-invasive ventilation (NIV), and longer duration from symptom onset to severe disease were protective against spontaneous pneumothorax (*P* < 0.001).

**Table 2:** Univariate regression analyses of risk factors for illness.

Variable	Classification criteria	Crude OR	95% CI	*P* value
Age (years)	≤ 40	1.0		
	40–60	0.307	0.104–0.905	***P* < 0.050**
	> 60	0.071	0.026–0.194	***P* < 0.001**
Sex	Male	1.0		
	Female	0.257	0.135–0.488	***P* < 0.001**
Comorbidities	No	1.0		
	Yes	0.241	0.135–0.432	***P* < 0.001**
Smoking status	No	1.0		
	Yes	0.960	0.391–2.359	*P* = 0.929
Respiratory rate	≤ 30	1.0		
	> 30	8.085	3.263–20.033	***P* < 0.001**
Oxygen saturation	≥ 93%	1.0		
	< 93%	1.764	0.969–3.211	*P* = 0.063
WBC (× 10^9^/L)		0.987	0.954–1.021	*P* = 0.443
Lymphocyte (× 10^9^/L)		1.224	0.755–1.983	*P* = 0.412
C-reactive protein (mg/L)	< 20	1.0		
	≥ 20	1.160	0.630–2.137	*P* = 0.633
Respiratory support	COT	1.0		
	HFNC	0.171	0.087–0.337	***P* < 0.001**
	NIV	0.125	0.060–0.261	***P* < 0.001**
Time from symptom onset to severe status	≤ 7	1.0		
	> 7	0.213	0.118–0.384	***P* < 0.001**

Bold is that the *P*-value represents significant difference, i.e., *P<0.05*.

The following characteristics were all associated with an increased risk of death: advanced age, comorbidities, types of pneumothorax/pneumomediastinum, NIV before SP/P, and treatment methods (*P* < 0.05).

### Multivariate logistic regression analysis and restricted cubic splines (RCS)

**Figure 2:**
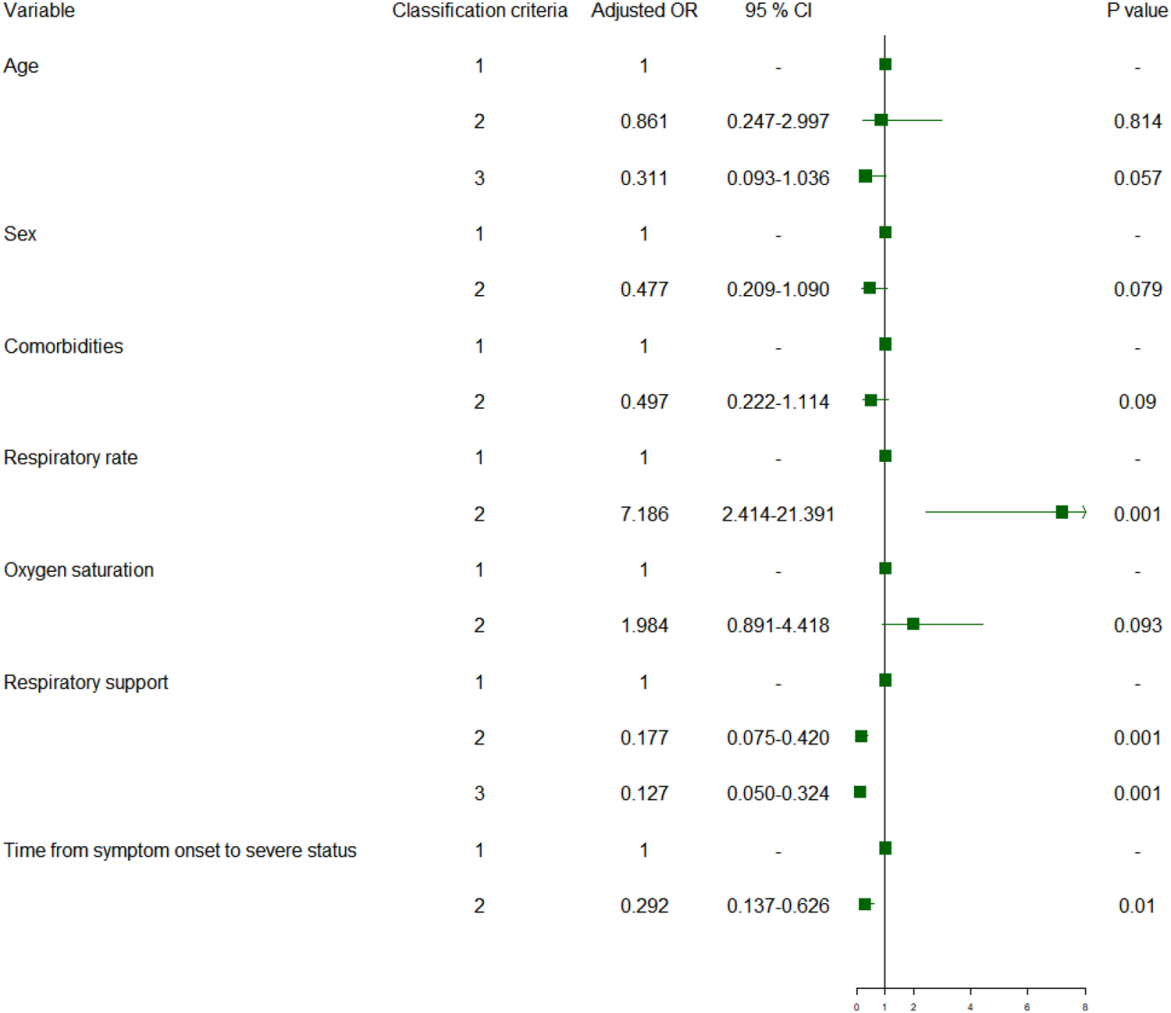
Multivariate regression analyses of risk factors for illness. (Age: ≤ 40 = 1; 40–60 = 2; > 60 = 3; Sex: Male = 1; Female = 2; Comorbidities : No = 1; Yes = 2; Respiratory rate: ≤ 30 = 1; > 30 = 2; Oxygen saturation: ≥ 93% = 1; < 93% = 2; Respiratory support: COT = 1; HFNC = 2; NIV = 3; Time from symptom onset to severe status: ≤ 7 = 1; > 7 = 2.).

Multivariate logistic regression analyses were performed (Fig. 2 and Supplementary Table 4) to identify the risk factors of illness and death in patients with COVID-19 and SP/P. The results showed that the odds ratio of SP/P were 7.186 in patients with a respiratory rate higher than 30 BPM (95% CI 2.414–21.391, *P* < 0.001). Longer duration from symptom onset to severe disease and early non-invasive respiratory support were protective against SP/P (*P* < 0.01). However, patients with delayed intubation due to early application of HFNC or NIV had a higher mortality rate when they experienced SP/P (*P* < 0.05). Additionally, advanced age increased the risk of death (*P* < 0.05).

**Figure 3:**
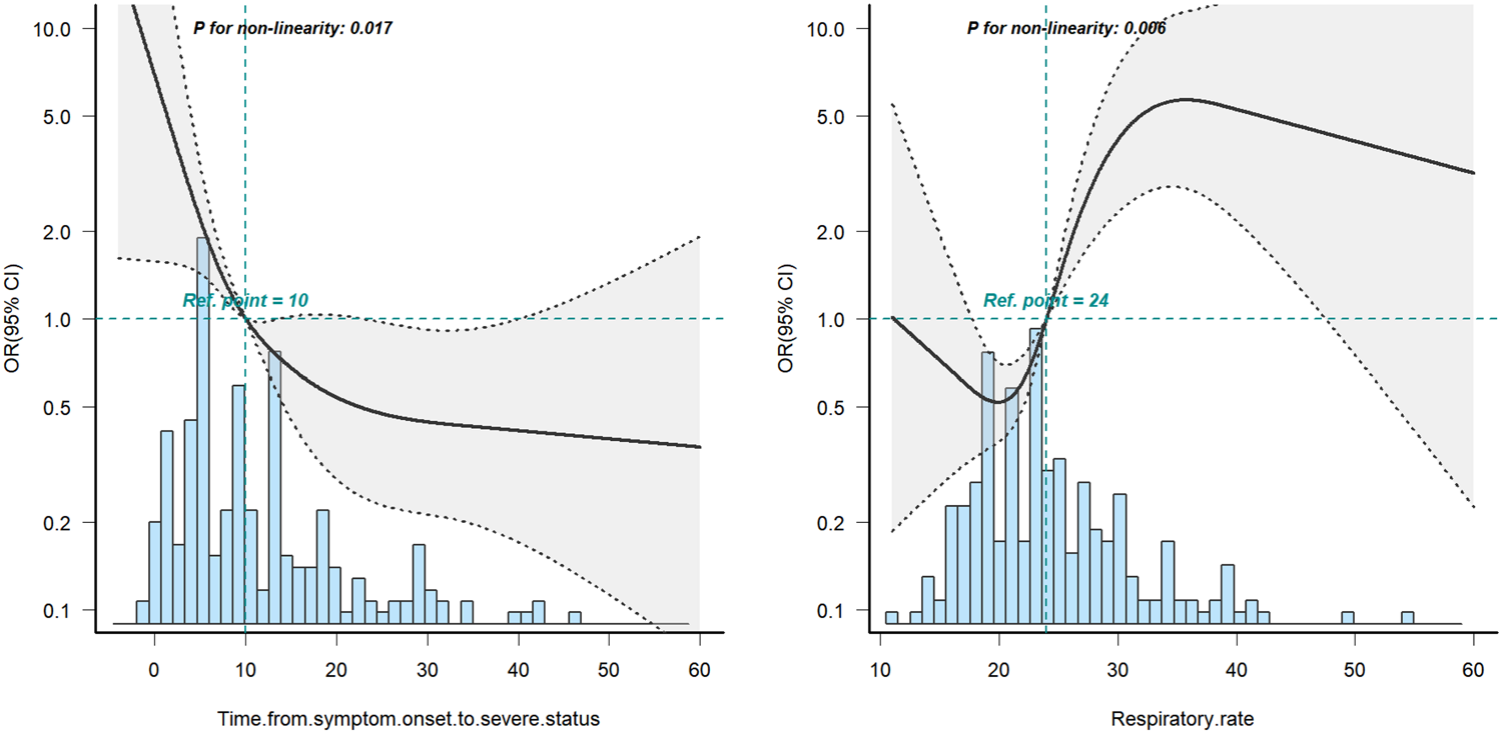
Restricted cubic splines (RCS). Respiratory rate and the time from symptom onset to severe disease had nonlinear relationships with the occurrence of SP/P.

Then, restricted cubic splines (RCS) were used to describe the nonlinear relationship between variables and outcomes (Fig. 3). The data showed that respiratory rate and the time from symptom onset to severe disease had nonlinear relationships with the occurrence of SP/P (*P* < 0.001).

### The relationship between death and SP/P

We used four different logistic regression models to analyze the relationship between death risk and SP/P (Table 3).

**Table 3:** The relationship between death and spontaneous pneumothorax/pneumomediastinum.

Variable	Classification criteria	Model 1			Model 2			Model 3			Model 4		
		Adjusted OR	95% CI	*P* value	Adjusted OR	95% CI	*P* value	Adjusted OR	95% CI	*P* value	Adjusted OR	95% CI	*P* value
SP/P	No	1.0			1.0			1.0			1.0		
	Yes	0.617	0.354–1.076	*P* = 0.089	0.967	0.505–1.851	*P* = 0.919	1.662	0.696–3.965	*P* = 0.252	2.047	0.726–5.775	*P* = 0.176

Model 1: we did not adjust any confounding factors.

Model 2: we adjusted age, gender, comorbidities, smoking status.

Model 3: we adjusted age, gender, comorbidities, smoking status, oxygen saturation, respiratory rate, respiratory support.

Model 4: we adjusted for model 3 plus systolic pressure, diastolic pressure, pulse, temperature, WBC, lymphocyte, C-reactive protein, time from symptom onset to severe status.

Without adjusting for any confounding factors, model 1 showed that SP/P was not a risk factor for death among patients with severe and critical COVID-19 (OR 0.617, 95% CI 0.354–1.076).

Using model 2 and after adjusting for age, gender, comorbidities, and smoking status, the data showed that the odds of death were 0.967 in COVID-19 patients who experienced SP/P (95% CI 0.505–1.851, *P* 0.919).

Model 3 was adjusted for age, gender, comorbidities, smoking status, oxygen saturation, respiratory rate, and respiratory support. Based on model 3, SP/P increased the risk of death (OR 1.662, 95% CI 0.696–3.965).

In addition to several confounders in model 3, model 4 was adjusted for systolic blood pressure, diastolic blood pressure, pulse rate, temperature, WBC count, lymphocyte count, C-reactive protein, and time from symptom onset to severe disease. The data showed that the odds of death was 2.047 in patients with SP/P (95% CI 0.726–5.775, *P* = 0.176) compared with those without SP/P.

## Discussion

Although SP/P was a rare complication of COVID-19, it can aggravate the severe condition and lead to poor prognosis^13^. P-SILI is often mixed with primary viral infection and it is difficult to make an early diagnosis in clinical practice. We believed that SP/P is the most severe complication of P-SILI. This study confirmed that P-SILI can markedly aggravate the primary disease. Therefore, it is necessary to further detect the clinical characteristics and risk factors of P-SILI-associated pneumothorax/pneumomediastinum.

This study confirmed that a respiratory rate higher than 30 BPM is a risk factor for SP/P. Patients with severe COVID-19 often had increased respiratory drive, as evidencd by increased tidal volume and respiratory rate^14^. For patients who do not receive ventilator-assisted respiration, clinicians cannot accurately and continuously monitor and record their tidal volume. The monitor can display the respiratory rate of patients with severe disease in a real-time manner. Therefore, it can be speculated that patients with COVID-19 have increased tidal volume only when they have increased respiratory rate. Meanwhile, a large number of studies have confirmed that the relationship between increased respiratory drive and lung injury. Mascheroni et al.^15^ have observed that prolonged hyperventilation during the infusion of sodium salicylate into the cisterna magna of spontaneously breathing adult sheep resulted in acute lung injury. Retamal and colleagues^16^ showed that marked inspiratory swing pressure led to heterogeneity in the distribution of ventilation and aggravates lung injury. Early but not late mechanical ventilation can prevent P-SILI. Therefore, COVID-19 patients with increased respiratory rate should be promptly treated to reduce P-SILI.

This study confirmed that prolonged disease was a protective factor for COVID-19 patients. Patients with early-onset ARDS are at higher risk of pneumonia, septic shock, lower oxygenation saturation and higher APACHE II score^17^; thus, they are more likely to develop SP/P. In addition, this study found that early non-invasive respiratory support was a protective factor because of improved oxygenation and reduced respiratory workload^18,19^. In addition, NIV can directly display tidal volume and the physician will be vigilant once the patient has increased tidal volume.

The early use of non-invasive respiratory support for COVID-19 patients indicated that they had poor oxygenation; therefore, we speculate that these patients already had increased respiratory drive. In addition, tachypnea is a risk factor for HFNC/NIV failure^20^. Therefore, if the patient developed tachypnea before HFNC/NIV, mechanical ventilation can easily lead to pneumothorax/pneumomediastinum^21^, and deteriorate patient’s oxygenation and prognosis^13^. This may explain why COVID-19 patients who receive early non-invasive ventilation have a higher mortality rate after experiencing SP/P.

Mechanical ventilation cannot effectively improve the prognosis of patients if COVID-19 co-exists with SP/P because positive pressure of mechanical ventilation can exacerbate pneumothorax and even cause barotrauma and air leak^22^. Moreover, patients with SP/P have a higher risk of death. CURB-65 score suggests that advanced age is a risk factor for death in pneumonia^23–25^.

This study confirmed that SP/P was a risk factor for death in patients with severe COVID-19 (OR 2.047). P-SILI seriously damages pulmonary structure and function; therefore, the possibility of pulmonary rehabilitation greatly reduces. Unfortunately, there was no significant difference (*P* = 0.176) in our study, which may be related to two reasons. First, our sample size was small. Second, most of the participants in the control group came from Huoshenshan hospital, where patients had more severe diseases.

The main limitation of our study was its retrospective nature. Additionally, most of our data were from the existing literature, and some details were missing. Therefore, a large-scale prospective study is warranted to further determine the risk factors of illness and death in patients with COVID-19 who have been complicated with SP/P.

## Conclusion

Our case-control study confirmed the existence of P-SILI in severe COVID-19 with acute respiratory failure. Increased respiratory rate is a risk factor for P-SILI. P-SILI-associated pneumothorax/pneumomediastinum is a risk factor for worse outcomes of COVID-19. It is necessary to further explore the indicators of P-SILI.

### Supplementary Information

Supplementary Tables.

## Data Availability

The datasets used and/or analyzed during this study are available from the corresponding author on reasonable request.

## Abbreviations

*COVID-19*: Coronavirus disease 2019
*ARDS*: Acute respiratory distress syndrome
*VILI*: Ventilator-induced lung injury
*P-SILI*: Patient self-inflicted lung injury
*ECMO*: Extracorporeal membrane oxygenation
*DAD*: Diffuse alveolar damage
*SP/P*: Spontaneous pneumothorax/pneumomediastinum
*WBC*: White blood cell
*HFNC*: High-flow nasal cannula
*NIV*: Non-invasive ventilation
*MV*: Mechanical ventilation
