# Supplementary material for: Patient self-inflicted lung injury associated pneumothorax/pneumomediastinum is a risk factor for worse outcomes of severe COVID-19: a case-control study
Source: Sci Rep. 2024 Jul 4;14:15437. doi: 10.1038/s41598-024-66229-0 (PMC11224394; doi:10.1038/s41598-024-66229-0)
Supplement: Supplementary file 1 — Supplementary Tables. [file 41598_2024_66229_MOESM1_ESM.docx]

**Supplementary Table 1** Basic information of recruited literatures.

| **Authors** | **Country** | **publication**  **year** | **Authors** | **Country** | **publication**  **year** | **Authors** | **Country** | **publication**  **year** | **Authors** | **Country** | **publication**  **year** |
| --- | --- | --- | --- | --- | --- | --- | --- | --- | --- | --- | --- |
| KONG N, et al. | Hangzhou, China | Aug. 2020 | HAZARIWALA V, et al. | Dearborn, MI, USA | Oct. 2020 | CHEN X, et al. | Wuhan, China | Sep. 2020 | UCPINAR B A, et al. | Istanbul, Turkey | Jun. 2020 |
| WANG W, et al. | Hangzhou, China | Aug. 2020 | ZHOU C, et al. | Wuhan, China | Apr. 2020 | GILLESPIE M, et al. | Philadelphia, PA, USA | Sep. 2020 | HAMEED M, et al. | Doha, Qatar | Oct. 2020 |
| OYE M, et al. | Jacksonville, Fl, USA | Nov. 2020 | MOHAN V, et al. | Trenton, New Jersey, USA | May. 2020 | SONIA F, et al. | Bronx, USA | Aug. 2020 | TUCKER L, et al. | Orlando, FL, USA | Nov. 2020 |
| REHMAN T, et al. | Oak Lawn, IL | Oct. 2020 | CAVIEZEL C, et al. | Switzerland | Sep. 2020 | MIMOUNI H, et al. | Oujda, Morocco | Oct. 2020 | KOLANI S, et al. | Fez, Morocco | May. 2020 |
| AHMED I, et al. | Karachi, Pakistan | Jan. 2021 | HUIS IN 'T VELD M A, et al. | Tilburg, the Netherlands | Jun. 2021 | DIAZ A, et al. | USA | Mar. 2021 | BOZAN Ö, et al. | Mersin, Turkey | Sep. 2021 |
| JANSSEN J, et al. | Venlo, the Netherlands | Feb. 2021 | NALEWAJSKA M, et al. | Szczecin, Poland | Feb. 2021 | BUONSENSO D, et al. | Rome, Italy | Jun. 2021 | MARSICO S, et al. | Barcelona, Spain | Jan. 2021 |
| ALAVIAN N, et al. | Chapel Hill, NC, USA | Sep. 2021 | GUTIERREZ-ARIZA J C, et al. | Cartagena, COL | Oct. 2021 | RASHEDI S, et al. | Tehran, Iran | May. 2021 | MARWAH V, et al. | Pune, India | Jul. 2021 |
| KABI A, et al. | Rishikesh, IND | Sep. 2021 | REANDELAR M J, et al. | Jamaica, USA | Nov. 2021 | SURESH K, et al. | Johnstown, USA | Nov. 2021 | ELKATTAWY S, et al. | Elizabeth, USA | Apr. 2021 |
| CHERIAN A, et al. | Madhya Pradesh, India | Jun. 2021 | ESSA R A, et al. | Kurdistan Region, Iraq | Jul. 2021 | ABBAS M, et al. | Aleppo, Syria | Dec. 2021 | IUORIO A, et al. | Italy | Sep. 2021 |
| JATOI T A, et al. | Karachi, Pakistan | Jan. 2021 | ARMILLAS-CANSECO F, et al. | México City, México | Jun. 2021 | MOUSSA N, et al. | CHU Hedi Chaker Sfax | Jan. 2021 | TATA R, et al. | Hyderabad, IND | Jun. 2021 |
| ANJUM I, et al. | Multan, Punjab, Pakistan | Nov. 2021 | RAFIEE M J, et al. | Tehran, Iran | Mar. 2021 | UMAR SHAHZAD M, et al. | Lancaster LA1 4RP, UK | Jan. 2021 | THEIN O S, et al. | Birmingham, UK | Mar. 2021 |

Continued

| **Authors** | **Country** | **publication**  **year** | **Authors** | **Country** | **publication**  **year** | **Authors** | **Country** | **publication**  **year** | **Authors** | **Country** | **publication**  **year** |
| --- | --- | --- | --- | --- | --- | --- | --- | --- | --- | --- | --- |
| DOĞAN B, et al. | İzmir, Türkiye | Mar. 2021 | FARAH R, et al. | Safed, Israel | May. 2022 | REYES S, et al. | TX, United States | Jun. 2022 | BLONDEAU-LECOMTE E, et al. | Gainesville, FL, USA | Jul. 2022 |
| CORAPLI G, et al. | Batman, Turkey | Apr. 2022 | NUR MUNIRAH I, et al. | Malaysia | Jan. 2022 | CARERJ M L, et al. | Frankfurt am Main, Germany | Jan. 2022 | YASSIN Z, et al. | Iran | Feb. 2022 |
| PACHECO-MONTOYA D, et al. | Ecuador | Mar. 2022 | LOVE J, et al. | Chicago, USA | Mar. 2022 | FAN Q Q, et al. | Wuhan, China | Oct. 2020 | SUN R H, et al. | Wuhan, China | May. 2020 |
| WANG J, et al. | Wuhan, China | May. 2020 | DE ALBUQUERQUE J H C, et al. | Fortaleza, CE, Brasil | Mar. 2021 | KUHAJDA I, et al. | Serbia | May. 2021 | NOVKOVIC L, et al. | Kragujevac, Serbia | May. 2021 |
| SHAN S, et al. | Xiangyang, China | Oct. 2020 | ALHAKEEM A, et al. | Doha, Qatar | Sep. 2020 | QUINCHO-LOPEZ A, et al. | Lima, Peru | Sep. 2020 | ELHAKIM T S, et al. | Florida, USA | Dec. 2020 |
| YOUNES I, et al. | Elizabeth, USA | Dec. 2020 | URIGO C, et al. | Sassari, Italy | Dec. 2020 | HUA D T, et al. | Torrance, CA, USA | Apr. 2021 | CHOWDHARY A, et al. | New Delhi, India | Nov. 2021 |
| MOHAMMADI A, et al. | Tabriz, Iran | Feb. 2021 | JOSEPH V M, et al. | Shillong, Meghalaya, India | Dec. 2021 | AL ARMASHI A R, et al. | USA | Dec. 2021 | JACQMIN G, et al. | Tournai, Belgium | Nov. 2021 |
| ATA F, et al. | Doha, Qatar | May. 2022 |  |  |  |  |  |  |  |  |  |

**Supplementary Table 2** Demographics and clinical characteristics of spontaneous pneumothorax/pneumomediastinum patients.

| **Clinical variable** | **Total cases**  **(N= 121)** | **Group 1**  **(N= 64)** | **Group 2**  **(N= 38)** | **P value** |
| --- | --- | --- | --- | --- |
| Age (years) mean ± SD | 52.02±16.551 | 49.42±17.864 | 56.29±13.994 | ***P*<0.050** |
| Sex |  |  |  | *P*=0.163 |
| Male (%) | 86.0% | 89.1% | 78.9% |  |
| Female (%) | 14.0% | 10.9% | 21.1% |  |
| Comorbidities |  |  |  | ***P*<0.010** |
| No (%) | 52.9% | 60.9% | 31.6% |  |
| Yes (%) | 47.1% | 39.1% | 68.4% |  |
| Smoking status |  |  |  | *P*=0.408 |
| No (%) | 90.9% | 93.8% | 86.8% |  |
| Yes (%) | 9.1% | 6.3% | 13.2% |  |
| Oxygen saturation☆ |  |  |  | *P*=0.206 |
| ≥93% (%) | 21.5% | 23.4% | 13.2% |  |
| <93% (%) | 78.5% | 76.6% | 86.8% |  |
| Respiratory rate☆ |  |  |  | *P*=0.099 |
| ≤30 (%) | 58.7% | 64.1% | 47.4% |  |
| >30 (%) | 41.3% | 35.9% | 52.6% |  |
| Diagnosis |  |  |  | *P*=0.074 |
| Pneumomediastinum (%) | 43.8% | 48.4% | 39.5% |  |
| Pneumothorax |  |  |  |  |
| Unilateral (%) | 24.0% | 29.7% | 15.8% |  |
| Bilateral (%) | 9.9% | 7.8% | 10.5% |  |
| Both (%) | 22.3% | 14.1% | 34.2% |  |
| Respiratory support before SP/P |  |  |  | ***P*<0.001** |
| Conventional oxygen therapy (%) | 67.8% | 82.8% | 44.7% |  |
| Noninvasive respiratory support (%) | 32.2% | 17.2% | 55.3% |  |
| Treatment |  |  |  | ***P*=0.001** |
| Conservative treatment (%) | 33.9% | 46.0% | 19.4% |  |
| Escalation of respiratory support (%) | 21.7% | 14.3% | 33.3% |  |
| Drainage (%) | 30.4% | 33.3% | 19.4% |  |
| Both (%) | 13.9% | 6.3% | 27.8% |  |
| The time interval from symptom onset to SP/P (days) mean ± SD☆ | 15.60±9.815 | 16.44±10.491 | 13.89±5.816 | *P*=0.119 |

**Note:** “☆” indicates that these indicators are subject to missing value analysis.

**Supplementary Table 3** Univariate regression analyses of risk factors for death.

| **Variable** | **Classification criteria** | **Crude OR** | **95 % CI** | **P value** |
| --- | --- | --- | --- | --- |
| Age (years) | ≤40 | 1.0 |  |  |
|  | 40-60 | 3.935 | 1.296-11.947 | ***P*<0.050** |
|  | >60 | 2.976 | 0.980-9.034 | *P*=0.054 |
| Sex | Male | 1.0 |  |  |
|  | Female | 2.171 | 0.718-6.566 | *P*=0.170 |
| Comorbidities | No | 1.0 |  |  |
|  | Yes | 3.380 | 1.447-7.897 | ***P*<0.010** |
| Smoking status | No | 1.0 |  |  |
|  | Yes | 2.273 | 0.571-9.049 | *P*=0.244 |
| Oxygen saturation | ≥93% | 1.0 |  |  |
|  | <93 | 2.020 | 0.670-6.095 | *P*=0.212 |
| Respiratory rate | ≤30 | 1.0 |  |  |
|  | >30 | 1.981 | 0.876-4.480 | *P*=0.101 |
| Diagnosis | Pneumomediastinum | 1.0 |  |  |
|  | Unilateral pneumothorax | 0.653 | 0.216-1.972 | *P*=0.449 |
|  | Bilateral pneumothorax | 1.653 | 0.387-7.063 | *P*=0.497 |
|  | Both | 2.985 | 1.045-8.530 | ***P*<0.050** |
| Respiratory support before SP/P | COT | 1.0 |  |  |
|  | HFNC | 7.482 | 2.305-24.293 | ***P*=0.001** |
|  | NIV | 4.676 | 1.454-15.046 | ***P*=0.010** |
| Treatment | Conservative treatment | 1.0 |  |  |
|  | Escalation of respiratory support | 5.524 | 1.672-18.250 | ***P*<0.010** |
|  | Drainage | 1.381 | 0.421-4.533 | *P*=0.595 |
|  | Both | 10.357 | 2.495-42.990 | ***P*=0.001** |
| The time interval from symptom onset to SP/P | ≤7 | 1.0 |  |  |
|  | >7 | 1.359 | 0.469-3.938 | *P*=0.571 |

**Supplementary Table 4** Multivariate regression analyses of risk factors for death.

| **Variable** | **Classification criteria** | **Model 1** | | |  | **Model 2** | | |  | **Model 3** | | |
| --- | --- | --- | --- | --- | --- | --- | --- | --- | --- | --- | --- | --- |
|  |  | **Adjusted OR** | **95% CI** | **P value** |  | **Adjusted OR** | **95% CI** | **P value** |  | **Adjusted OR** | **95% CI** | **P value** |
| Diagnosis | Pneumomediastinum | 1.0 |  |  |  |  |  |  |  |  |  |  |
|  | Unilateral pneumothorax | 0.735 | 0.109-4.966 | *P*=0.752 |  |  |  |  |  |  |  |  |
|  | Bilateral pneumothorax | 1.320 | 0.155-11.259 | *P*=0.799 |  |  |  |  |  |  |  |  |
|  | Both | 1.721 | 0.377-7.851 | *P*=0.483 |  |  |  |  |  |  |  |  |
| Comorbidities | No | 1.0 |  |  |  | 1.0 |  |  |  |  |  |  |
|  | Yes | 1.519 | 0.447-5.161 | *P*=0.503 |  | 1.687 | 0.538-5.293 | *P*=0.370 |  |  |  |  |
| Age (years) | ≤40 | 1.0 |  |  |  | 1.0 |  |  |  | 1.0 |  |  |
|  | 40-60 | 3.562 | 0.746-17.001 | *P*=0.111 |  | 3.513 | 0.758-16.279 | *P*=0.108 |  | 4.569 | 1.091-19.144 | ***P*<0.050** |
|  | >60 | 4.524 | 0.824-24.824 | *P*=0.082 |  | 4.465 | 0.903-22.082 | *P*=0.067 |  | 5.953 | 1.361-26.032 | ***P*<0.050** |
| Respiratory support before SP/P | COT | 1.0 |  |  |  | 1.0 |  |  |  | 1.0 |  |  |
|  | HFNC | 6.647 | 1.590-27.789 | ***P*<0.010** |  | 5.759 | 1.463-22.670 | ***P*<0.050** |  | 6.281 | 1.630-24.209 | ***P*<0.010** |
|  | NIV | 5.628 | 1.151-27.522 | ***P*<0.050** |  | 6.430 | 1.424-29.020 | ***P*<0.050** |  | 6.609 | 1.486-29.392 | ***P*<0.050** |
| Treatment | Conservative treatment | 1.0 |  |  |  | 1.0 |  |  |  | 1.0 |  |  |
|  | Escalation of respiratory support | 3.768 | 0.904-15.706 | *P*=0.069 |  | 4.783 | 1.216-18.817 | ***P*<0.050** |  | 4.845 | 1.229-19.107 | ***P*<0.050** |
|  | Drainage | 1.156 | 0.213-6.267 | *P*=0.866 |  | 1.250 | 0.325-4.801 | *P*=0.746 |  | 1.272 | 0.335-4.829 | *P*=0.723 |
|  | Both | 19.011 | 2.248-160.742 | ***P*<0.010** |  | 21.241 | 3.525-127.997 | ***P*=0.001** |  | 22.092 | 3.658-133.420 | ***P*=0.001** |
